# Chondroitin Sulfate, Hyaluronic Acid and Chitin/Chitosan Production Using Marine Waste Sources: Characteristics, Applications and Eco-Friendly Processes: A Review

**DOI:** 10.3390/md11030747

**Published:** 2013-03-11

**Authors:** José Antonio Vázquez, Isabel Rodríguez-Amado, María Ignacia Montemayor, Javier Fraguas, María del Pilar González, Miguel Anxo Murado

**Affiliations:** 1 Group of Recycling and Valorisation of Waste Materials (REVAL), Marine Research Institute (IIM-CSIC), r/Eduardo Cabello, 6. Vigo, Galicia 36208, Spain; E-Mails: sabelara@iim.csic.es (I.R.-A.); rvrlab@iim.csic.es (J.F.); pgonzalez@iim.csic.es (M.P.G.); reciclaa@iim.csic.es (M.A.M.); 2 Research Centre of Vine and Wine Related Science (ICVV-CSIC), Scientific and Technical Complex of the University of La Rioja, Logroño 26006, Spain; E-Mail: nachi@iim.csic.es

**Keywords:** glycosaminoglycans, by-products upgrading, chondroitin sulphate, hyaluronic acid, chitin and chitosan, eco-friendly processes, clean production

## Abstract

In the last decade, an increasing number of glycosaminoglycans (GAGs), chitin and chitosan applications have been reported. Their commercial demands have been extended to different markets, such as cosmetics, medicine, biotechnology, food and textiles. Marine wastes from fisheries and aquaculture are susceptible sources for polymers but optimized processes for their recovery and production must be developed to satisfy such necessities. In the present work, we have reviewed different alternatives reported in the literature to produce and purify chondroitin sulfate (CS), hyaluronic acid (HA) and chitin/chitosan (CH/CHs) with the aim of proposing environmentally friendly processes by combination of various microbial, chemical, enzymatic and membranes strategies and technologies.

## 1. Introduction

The world capture of marine organisms including aquaculture (mainly fish, mollusks and crustaceans) amounts to 132 million tons [1]. Among them, more than 35% of the total weight is handled as by-product and waste that include animal fractions (skeletons, heads, viscera) generated in seafood production or species, sizes or qualities without commercial value (discards and by-catch). Commonly, the production of such wastes is located in coastline areas with the corresponding problem associated with environmental pollution generated by an inefficient residue management [2,3]. In addition, the overexploitation of several species (e.g., sharks) has led to ecological risks derived from the reduction of biological resources [4]. To establish a more efficient control of fisheries, to increase the profitability of seafood operations and to satisfy environmental regulations, low cost and environmentally friendly technologies are being evolved by the necessity of recovering all the materials (polysaccharides, proteins, oils, minerals) [3,5,6,7]. Recently, alternative methods have been developed to obtain different products and molecules: enzymes, glycosaminoglycans, chitin, gelatin, biosilage, marine peptones, *etc.*, from skeletons, skins, viscera, heads, *etc.* [8,9,10,11,12,13,14,15,16,17,18,19,20,21,22]. Bearing in mind the number of applications and the economical value of final products, glycosaminoglycans and chitin are two of the most important and relevant compounds to upgrade from marine wastes [23].

Glycosaminoglycans (GAGs) are heteropolysaccharides defined by a repeating disaccharide unit without branched chains in which one of the two monosaccharides is always an amino sugar (*N*-acetylgalactosamine or *N*-acetylglucosamine) and the other one is a uronic acid. They are present on all animal cell surfaces and in the extracellular matrix where are known to bind and regulate different proteins (e.g., growth factors, enzymes, cytokines). After purification, they are used in numerous contexts from food, cosmetic and clinical areas [24,25,26].

Chemical structure of chitin is also a long linear chain formed by successive units of an amino monosaccharide (*N*-acetylglucosamine); however, it is not commonly classified as GAGs. It is the second most extended polysaccharide in nature after cellulose, forming part of microorganism cell walls, exoskeleton of insects and shells of crustaceans. Both chitin and its partially deacetylated form chitosan have been intensely studied in recent years with a promising potential for applications in pharmacy, alimentary and biomedicine devices [27,28,29].

Several methodologies have been developed to produce the mentioned biopolymers prepared with steps of hydrolysis and purification that are usually expensive and/or environmentally not friendly, for instance, to manage large volumes of alkalis and strong acids needed in hydrolysis and to use specific chromatographic techniques hardly scale-up in purification. The present review addresses an overview of different sustainable and clean processes to recover chondroitin sulfate (CS), hyaluronic acid (HA) and chitin/chitosan (CH/CHs) from marine waste materials.

## 2. Glycosaminoglycans

Traditionally, the production of GAGs is obtained from mammalian tissues mainly generated in slaughterhouse: Rooster combs, cartilage (tracheas and nasal from bovine and swine) and umbilical cords. However, as a consequence of the concern due to the bovine spongiform encephalopathy (BSE) and other food chain crisis, the exploration of microorganism and marine organisms as source of those glycoconjugates has received increasing attention. Marine organisms like sponges, sea cucumbers, squids, mollusks, invertebrates and mainly cartilaginous material from fishes (shark, salmon, ray, *etc.*) are well-documented as potential producers of them [30,31,32].

Cartilage is a tissue formed by a matrix of collagen associated with proteoglycans, macromolecules with a core protein to which the GAGs chondroitin sulfate, keratan sulfate, dermatan sulfate and heparan sulfate are covalently attached by means of a trisaccharide linked to a serine residue. HA is the only non-sulfated GAGs and is not covalently bound to the protein in any tissue, although specific HA-protein interaction is shown [33]. CS and HA are the most valued GAGs in market because of its abundance in mammalian tissues, physiological functions and high activity.

### 2.1. Characteristics and Applications of CS

CS is formed by only one type of repeating disaccharide units of glucuronic acid (GlcA) and *N*-acetylgalactosamine (GalNAc) linked by β-(1→3) glycosidic bonds and sulfated in different carbon positions (CS no-sulfated is CS-O). The classification and type of CS is dependent on sulfate group placing: carbon 4 (CS-A), 6 (CS-C, more common), both 4 and 6 (CS-E), positions 6 of GalNAc and 2 of GlcA (CS-D) and 4 of GalNAc/2 of GlcA (CS-B) [34]. Moreover, the composition and concentration of CS depends on the function of the organism and tissue, thus, CS from terrestrial and marine sources contains diverse chain lengths and oversulfated disaccharides (shark, CS-D; dogfish, CS-A and CS-D; squid and salmon, CS-E; crocodile, CS-E; chicken CS-A and CS-E; ray, CS-A and CS-C) [35,36] at different relative concentrations (e.g., 9% in shark fin and 14% in chicken keel). 

In all cases, CS is an essential component of extracellular matrix of connective tissues in which plays a central role in various biological processes, such as the function and elasticity of the articular cartilage, hemostasis and inflammation, regulation of cell development, cell adhesion, proliferation and differentiation [37]. The number of commercial applications has been continuously increased, due to its high biocompatibility, mainly in the engineering of biological tissues associated with the processes of bone repair, cartilage and cutaneous wound. Moreover, its combination with other biopolymers (such as collagen, proteoglycans and HA) to formulate scaffolds with slow and controlled biodegradability that promote and accelerate the regeneration of damaged structures has been studied [38,39]. In these injuries, CS is involved in reepithelialization, in the stimulation of neovascularization and supplying growth factors and cytokines when it is included in hydrogels [40,41].

Recent studies have demonstrated that CS-E is a potent antiviral [42] whereas CS-proteoglycan is a potential target for the development of vaccines against malaria [43]. New findings about the sulfation pattern of CS related with cancer cell mechanisms have been also reported [44]. This feature revealed its ability and potential role as biomarker to early detection of diverse types of cancer [45]. Furthermore, fucosylated CS (CS-F) was obtained from sea cucumber has led to excellent results to inhibit adenocarcinoma growth in lungs using mouse model [46]. On the other hand, partially purified CS is also used as food preservative with emulsifying properties [47]. Nevertheless, the most successful commercial products of CS, by market volume and benefits, are those associated with cartilage regeneration, anti-inflammatory activity and osteoarthritis [48,49]. In this way, low/medium-molecular weight CS (inferior to 20 kDa) is orally administered in nutraceutical formulations to treat and prevent the osteoarthritis due to its inhibitory capacity of cartilage degradative enzymes [50].

From a marine perspective, shark fins have been the most commonly used source of CS but the increasing price of this substrate together with the irrational and non-controlled exploitation of shark stocks, as well other ecological aspects has led to the shark fishery on the brick of extinction [4,51]. The skeleton of ray is another attractive source of CS but similar bad habits on the stocks regulation have been also reported [51,52].

### 2.2. Characteristics and Applications of HA

HA is a linear, high molecular weight unbranched and non-sulfated GAG made by alternating disaccharide units of *N*-acetyl-d-glucosamine and d-glucuronic linked by β-(1→3) and β-(1→4) glycosidic bonds. It is ubiquitously distributed in connective tissues where is a major structural component of intercellular matrix. It has a fundamental role in controlling tissue permeation and hydration, macromolecular transport between cells and bacterial invasiveness [33]. The presence of HA is especially important in the umbilical cord, rooster comb, synovial fluid, vitreous humor (VH) and cell wall of *Streptococci* bacteria [53]. It holds a large number of water molecules in its molecular domain and occupies enormous hydrodynamic space in solution [54]. This characteristic (“swelling property”) together with its chemical structure gives it a wide-ranging of physicochemical and biological properties and functions such as lubricity, viscoelasticity, biocompatibility, angiogenic and immunostimulatory. This polymer has great economical value with numerous applications in biotechnology, regenerative medicine and cosmetic fields such as plastic surgery, anti-aging cosmetics, arthritis treatment, joint injections, major burns and intra-ocular surgery [25,55]. The activity of HA is dependent on its size, hence all ranges of molecular weights are handled in specific usage area.

Originally, it has been obtained and commercialized from diverse mammalian substrates as rooster combs, synovial fluid, VH and umbilical cords [53]. Marine wastes have been also explored in the search of new sources of HA, being only found in VH of various fish species and in cartilage of chondrichthyes [56]. However, the most important alternative in recent years has been the development of microbial HA production by *Streptococcus* bacteria. This fermentation generates the best yields with higher concentrations of HA (>3 g/L) at lower costs and with more efficient downstream processes [57,58].

## 3. CS Production Processes

The types of applications for the formulations of CS or CS-derived, and therefore their market price, are dependent on the concentration and purity of this GAG in the commercial products. Different compounds including chemical solvents and detergents from isolation step and peptides, proteins, nucleic acids or organic compounds from tissues are commonly contaminating the samples; hence, they are reducing its commercial value and limiting its usage areas [49]. Clinical applications demand highly concentrated and pure CS in comparison with cosmetic, dietary supplements or food ingredients. Moreover, CS derived from fish (ray and shark) is referred as a better source than mammalian because of its sulfation pattern and safety. Therefore, it is especially important the development of highly yielded and low-cost extraction processes, maintaining the quality and great purity of CS in order to execute an optimum exploitation of marine sources.

In general, the methods of CS isolation from cartilage (the most interesting substrate from an industrial viewpoint) are defined for several years [59,60,61] and include various steps based on: (1) chemical hydrolysis of cartilage; (2) breakdown of proteoglycan core; (3) elimination of proteins and CS recovery; (4) purification of CS. The two first stages are mostly conducted by means of alkaline hydrolysis at high concentrations of NaOH, urea or guanidine HCl, subsequently combined with selective precipitation of GAG using cationic quaternary ammonium chemicals (as cetylpyridinium chloride), potassium thiocyanate, non-ionic detergents or alcoholic solutions [59,60], deproteinization by trichloroacetic acid and finally purification with gel filtration and/or ion-exchange and size-exclusion chromatography [62]. Unfortunately, those economically viable stages lead to unsatisfactory purity for clinical uses of CS. The techniques that improve final product quality need larger amounts of reagents and are time-consuming. In addition, costumers and manufactures try to develop more environmentally friendly and economical processes to obtain CS based on non contaminant solvent strategies.

**Figure 1:**
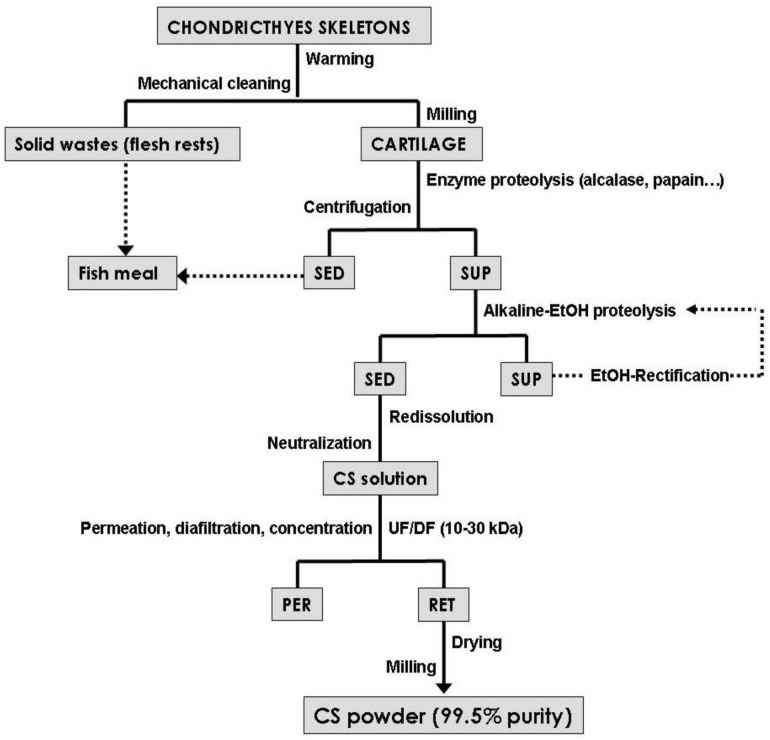
Overview of chondroitin sulfate (CS) recovery and purification processes from marine cartilage by-products. SED: sediment, SUP: supernatant, PER: permeate and RET: retentate.

Various alternative isolation methods have been recently developed to replace the classical methods for pursuing sustainability [63,64,65,66]. Those processes can be summarized as follows: digestion of cartilage and proteins mediated by enzymes, selective precipitations with alcoholic solutions, resuspension and neutralization with salt solutions and separation by molecular-weight using ultrafiltration-diafiltration technologies (UF-DF). Figure 1 shows a flow chart representing all the potential steps described for the downstream purification of marine CS. Firstly, the fishing by-products (e.g., ray skeletons or shark heads) are warmed separating the rests of flesh, excellent material for fish meal, and cartilage for CS production. Subsequently, dried and milled cartilage is hydrolyzed by proteases under controlled experimental conditions. Multiple enzymes have been studied, generally with successful results, with the objective of cartilage degradation, protein fraction breakdown and to obtain undamaged CS molecules. The proteolysis of proteoglycans from hammerhead shark fin cartilage was partially degraded by commercial papain but trypsin or superase were not effective [67]. Similar activity of papain digestion was also observed in adult zebrafish [68], ray [35] and dogfish tissues [69]. In all cases, the time of hydrolysis was superior to 18 h under optimal conditions of temperature (50–65 °C) and pH 7. Recently, a two-step enzymatic processing with alcalase and flavourzyme showed better yields of degradation with a significant reduction of time-processing [21]. Proteolytic and collagenolytic activities isolated from skate pancreas led to percentage of skate cartilage hydrolysis higher than 50% in 6 h [66]. The separation of hydrolysates is generally carried out by simple decantation or centrifugation removing the supernatant rich in CS and the rests of cartilage precipitated (useful as substrate for fish meal production). Tadashi [65] suggested a previous elimination stage of cartilage wastes based on the addition of activated charcoal at 55 °C.

The subsequent phase of alcoholic treatment is usually indicated by several authors as crucial for the selective precipitation of CS from the major protein presents in the hydrolysate [61,65,66,70]. The effectiveness of that process is dependent on the type and alcohol concentration and, in some cases, the influence of processing-temperature is also important [65]. Ethanol is the most commonly selected reagent for such precipitation, at concentrations of 40%–60%, due to its widespread use as a solvent of substances intended for human contact or consumption [65,66,70]. A recent report also proposed isopropanol at 40% for the purification of CS from scapular cartilage of shortfin mako shark [21]. In our lab, we have optimized the combination of alkaline proteolysis and selective precipitation of CS from ray cartilage by using alkaline hydroalcoholic solutions [66]. Under optimal conditions of NaOH 0.2 M and one volume of ethanol per volume of hydrolysate at room temperature with soft agitation for 1 h, more than 96% of CS recovery and CS purity were obtained. The repetition of this procedure, under the same experimental conditions, increased the CS purity up to 99%. The resuspension of CS sediment and pH neutralization is efficiently obtained by means of saline solutions as NaCl or sodium acetate, the salt excess can be subsequently removed by membrane dialysis method.

The last step of purification by membrane technologies is widely performed in the majority of the biomacromolecules downstream processing (with higher sizes of 1 kDa) because of its separation effectiveness, easy scale-up, cost effective device, numerous types and cut-off membranes and simple operatory and control. However, the use of described methodology, to remove low-molecular-weight materials and salts from neutralized or resupended CS solution, has been poorly studied. Large quantities of CS from salmon tissues were extracted by alkali treatment and subsequent purified by repeating UF procedure, demonstrating superior efficient than using ion exchange resin [71]. A two-step process based on enzyme extraction of CS and concentration-desalting by UF-DF was studied using skate cartilage as substrate and ceramic membranes [63]. The authors advised that the desalting step by DF should be improved with a higher filtering area due to the 40% remaining of salts in the final solution of CS. This inconvenience was improved via polyethersulfone membranes with molecular-weight cut-off at 10 kDa and 6 diavolume [66]. The UF-DF system was assembled with total recirculation to obtain CS of 99.6% purity at final concentration of 35–45 g/L. Higher CS concentrated liquid generated excessive viscosities that reduced the filtrate flow and filled the membranes.

Finally, the powder of CS can be obtained by drying the concentrated solutions without adding chemical solvents using spray dryer equipment or evaporation in an oven, depositing CS in thin layers on trays followed by milling performance.

### Microbial Production of CS

In order to avoid the health and ecological problems derived from the uses of mammalian and fishery wastes as substrate, different approximations to microbial production of CS-like polymers have been reported in recent years [37,72,73]. Initially, *Pasteurella multocida* was one of the bacteria selected as CS producer, but its well-known cholera pathogenicity has hindered and reduced its interest [74,75]. Excellent results were obtained in the production of capsular polysaccharide CS precursor (CSC) by *Escherichia coli* O5:K4:H4 under diverse experimental conditions and fermentation devices [37,73]. These authors have improved the CSC concentration of 0.2 g/L obtained in batch cultures [76] to 1.4 g/L with fed-batch operation [77] and more than 3 g/L using a membrane bioreactor [78]. Downstream processing finally yielded about 80% chondroitin with 90% purity [79]. Nevertheless, *E. coli* is a low virulent pathogen limiting its large scale production and CSC is an unsulphated structure of chondroitin (CS-O), with a furanose residue of fructose, which needs a subsequent step of chemical sulfation and hydrolysis of fructose monomer [80]. The production of CS by combined fermentation and chemical developments is a complementary alternative to achieve a global and sustainable control of chondrichthyes stocks. 

## 4. HA Production Processes

The most conventional materials employed for HA extraction are selected for its feasibility and concentration, thus, umbilical cord presents an average level of 4 g/L, synovial fluid from pig (3 g/L) or bovine (18 g/L) and rooster combs (25 g/L) [56,81,82,83]. Nevertheless, the risk of animal-derived pathogens, inter-species viral or prionic contaminations (e.g., BSE, epizootic aphtha) has obligated to explore and optimize other alternatives of production.

In marine organisms, the only clear source of HA is the VH present in the eyeball of fish species. VH volume and HA concentration is different depending on the selected fish, for instance, HA is obtained from eyeballs of shark and swordfish at 0.3 g/L from 18 mL of VH and 0.055 g/L from 70 mL, respectively [56]. HA is also present in the cartilage matrix in which is very important as structural element of the aggrecan in cartilaginous fishes; however, its relatively low content makes it economically unavailable for any industrial extraction process [84].

The traditional protocols for extraction of HA from animal substrates (e.g., rooster comb) are developed according to the works reported by Swann [85] and Balazs [86] that include the preparation of the material, washing with water or alcohol, aqueous or organic solvent (mainly chloroform) extraction, precipitation by cetylpiridinium chloride, filtering and successive extractions with chloroform, centrifugation and occasional chromatographic purification. Other authors indicated that both procedures are costly, laborious, time-consuming and lead to contaminated HA solutions that limit their applications in biopharma formulations [87]. 

**Figure 2:**
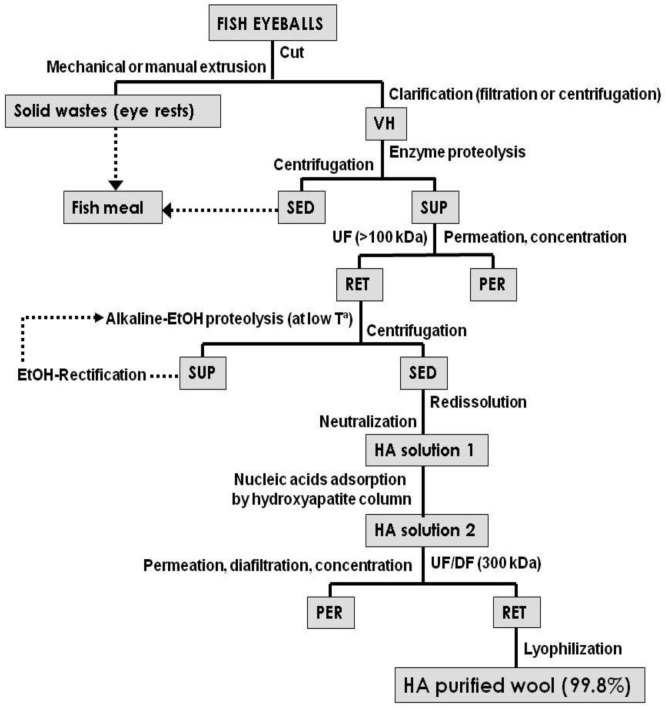
Flowchart of purification methods to extract hyaluronic acid (HA) from vitreous humor (VH) of fish eyeball. SED: sediment, SUP: supernatant, PER: permeate and RET: retentate.

Different strategies have been proposed for the extraction of HA from mammalian VH including deproteinization of bovine substrate with xylenesulphonate-Na [88], extraction with water, precipitation with ethanol and purification by DEAE-cellulose [89] and fractional precipitation using ethanol combined with enzymatic protein hydrolysis [59]. The most exhaustive method was applied to tuna eyeball substrate [90], performing precipitation with overcooled acetone, actinase digestion, thermal coagulation, dialization by membrane and cetylpyridinium chloride precipitation. Similarly, we have addressed a method to recover and purify HA of VH from various fish eyeballs (tuna, shark, and swordfish) using easier, faster and cheaper stages [56]: (1) initial clarification of VH extracted from frozen eyeball (using centrifugation or glass wool filtration); (2) enzyme proteolysis; (3) concentration by UF; (4) precipitation and alkaline proteolysis in hydroalcoholic medium at low temperature; (5) selective redissolution and neutralization; (6) separation, purification and concentration by UF-DF; (7) removing of nucleic acids using absorption with hydroxyapatite obtained from fish bone. Medical-grade purity of 99.9% (molecular weight 2000 kDa) was thus reached (Figure 2). The lyophilization of final solutions is an excellent lab resource to avoid physical degradation and size reduction but they are too expensive for industrial scale.

Although prices of vitreous HA are commercially high, economic viability is unclear given the cost of vitreous humor (VH) removal (expensive labor) and the overdependence on raw materials (opportunistic prices of fish eyes) that usually tends to the overexploitation of this marine resource. Microbial production easily contains the concentration equivalent (3 g/L) to 660 eyes of shark or 900 of swordfish. Additionally, the presence of blood in the eyes supply should be avoided as much as possible because the iron from haemoglobin degrades HA molecule.

### Microbial Production of HA on Marine Food Wastes

Bacterial production of HA using Lancerfield group A and C streptococci has been industrially developed to replace gradually the HA obtained from animal origin. Several culture variables have been studied and optimized such as lysozyme or hyaluronidase addition [91,92], agitation and aeration conditions [93,94,95], the type of bioreactor [96], effect of pH-gradient stress [97], continuous culture [98], medium optimization [99] and fed-batch operation [100]. Since no marine microorganisms have been discovered for HA production, the only marine approach for this bioproduction is derived from the substitution of commercial broths by alternative nutrients generated in the marine foodstuff manufacturing. Recently, the formulation of cultivation medium with mussel processing wastewaters (MPW), rich in glycogen as glucose substitutive, and peptones obtained from fish viscera by-products generated acceptable HA productions that were improved under fed-batch conditions [101,102]. Generally, HA downstream processes from post-incubated medium are easier than those reported for animal sources, especially if consumption of culture medium ingredients is complete at the end of fermentation. Cellular biomass precipitation (by means of detergent adding and centrifugation), deproteinization using proteases or specific adsorption to resin and membranes purification are the most conventional procedures. Other proposal includes silica gel filtration combined with active carbon treatment followed by diafiltration [58]. In Table 1, different alternatives and process conditions for CS and HA production from marine sources and some microbial cultivations are summarized.

**Table 1:** Summary of GAGs productionfrom marine sources (CS and HA), using marine culture broths for fermentation (HA) or by microbial fermentation (CS).

GAG	Type	Source	Process conditions	Yield ( *Y*)/Production (*P*) Purity (*Pu*)	Ref.
CS	CS-C	shark cartilage	proteolysis, alcoholic precipitation, membrane purification	*Y* = 57% (w/v)	[21]
CS	CS-A, CS-C	ray and shark cartilage	proteolysis, cetylpyridinium HCl and NaCl precipitations, filtration and dialization	*Y* = 10%–11% (w/v)	[35]
CS	CS-A, CS-C	skate fin	proteolysis, cetylpyridinium HCl precipitation, electrophoresis and cromatographic purification	-	[62]
CS	CS-A, CS-C	skate cartilage	proteolysis, purification (UF-DF)	-	[63]
CS	CS-A, CS-C	ray cartilage	proteolysis, alkaline-hydroalcoholic precipitation, purification (UF-DF)	*Y* = 15% (w/w)/*Pu* > 99%	[66]
CS	CS-A, CS-C	shark fin	proteolysis, guanidine HCl extraction, electrophoresis and cromatographic purification	*Y* = 84%	[67]
CS	CS-A, CS-C, CS-O	zebrafish cartilage	proteolysis, electrophoresis and cromatographic purification	-	[68]
CS	CS-A, CS-C, CS-D, CS-O	dogfish cartilage	proteolysis, alcoholic precipitation, cromatographic purification	*Y* = 5% (w/w)	[69]
CS	CS-A, CS-C, CS-O, CS-E	salmon nasal cartilage	proteolysis, alkaline hydrolysis, alcoholic precipitation, cation exchange separation	*Y* = 24% (w/w)/*Pu* = 99%	[70]
CS	CS-A, CS-C, CS-O, CS-E	salmon nasal cartilage	proteolysis, alkaline hydrolysis, alcoholic precipitation, purification (UF)	-	[71]
CS	CS-O	*E. coli* O5:K4:H4	batch operation	*P* = 0.2 g/L	[76]
CS	CS-O	*E. coli* O5:K4:H4	fed-batch operation	*P* = 1.4 g/L	[77]
CS	CS-O	*E. coli* O5:K4:H4	membrane bioreactor, fed-batch, purification (UF-DF)	*Y* = 80%/*P* = 3 g/L *Pu* = 90%	[78]
HA	-	shark HV	proteolysis, concentration (UF), selective precipitation, purification (UF-DF)	*P* = 0.3 g/L/*Pu* > 99.5%	[56]
HA	-	swordfish HV	proteolysis, concentration (UF), selective precipitation, purification (UF-DF)	*P* = 0.06 g/L/*Pu* > 99.5%	[56]
HA	-	*S. zooepidemicus*	medium: shark or ray peptones, fed-batch	*P* = 2.5 g/L	[101]
HA	-	*S. zooepidemicus*	medium: tuna peptones and MPW, batch	*P* = 2.5 g/L	[102]

## 5. Chitin and Chitosan

During recent years, CH and CHs have attracted a great interest due to their distinctive biological and physicochemical properties [28], which make them interesting polymers, among others, for biotechnology, medicine, cosmetics, food technology and textile applications.

Marine organisms are principal source of CH since it is a constituent of the organic matrix of the exoskeletons of arthropods such as crustaceans (crabs, lobsters and shrimps) and of the endoskeleton of mollusks [103]. Although CH can also be found in many other organisms including fungi [104], yeasts [105], algae and squid pen [106], the shell of marine crustaceans is the preferred source of CH due to their high availability as waste from the seafood processing industry [28].

However, traditional methods involved in the recovery of CH from shellfish are extremely hazardous, energy consuming and environmentally polluting, due to the need of using high amounts of mineral acid and alkali [107]. In addition, the deacetylation of CH to produce CHs requires the use of very intense alkaline treatments. Hence, alternative environmentally friendly processes are being assessed including the use of proteases or proteolytic bacteria for deproteinization and demineralization of crustacean shells [108,109]. Alternatively, fermentation using lactic acid bacteria (LAB) has been widely applied for the extraction of CH from crab [110,111] and shrimp [112,113] biowastes. In case of CHs production, fungus *Mucor rouxii* has been widely studied as an alternative source of chitosan.

### Characteristics and Applications of CH and CHs

CH is the most abundant biopolymer in nature after cellulose [27,28]. It is a linear polysaccharide composed of β-(1→4)-linked *N*-acetyl-d-glucosamine monomers. Its abundance in the environment is due to its role as major component in the supporting tissues of organisms such as crustacean, fungi and insects [114].

Depending on its source, CH can occur as either α, β or γ forms [115]. The α form has antiparallel microfibril orientation with strong intra and intermolecular hydrogen bonds and is the most abundant chitin in nature and the preferred form for industrial applications. The β-chitin form has parallel chains held by weak intra chain hydrogen bonds and occurs in squid pens [116]. A third and less characterized form, γ-chitin, has been described as a mixture of antiparallel and parallel chains, although there is controversy about the existence of this conformation [117,118].

Owing to the extensive hydrogen bonding in the solid state of α-chitin, it is insoluble in water, most organic acids and diluted acid and alkaline solutions [117]. However, it can be dissolved in concentrated hydrocloric, sulfuric and phosphoric acids as well as in dichloroacetic, trichloroacetic and formic acids [119]. In addition, special solvents such as hexafluoroacetone and *N*,*N*-dimethylacetamide containing 5%–8% lithium chloride have proved suitable for solubilizing CH [120]. Unlike α-chitin, β-chitin generally shows better solubility in most acids and swells in water considerably [28]. On the other hand, CHs is insoluble in either organic solvents or water [28], but it is soluble in diluted acid solutions below pH 6.0, due to the presence of free amino groups with a p*K*a value of 6.3 [121].

The lack of solubility of CH makes it necessary to modify the molecule for most of its applications. Among various reactions that can disrupt intra- and inter-molecular hydrogen bonds without cleaving glucosidic linkages, *N*-deacetylation is the simplest modification, which transforms CH to CHs [117]. The degree of *N*-acetylation (DA), *i.e*., the ratio of 2-acetamido-2-deoxy-d-glucopyranose to 2-amino-2-deoxy-d-glucopyranose structural units has a remarkable effect on CH solubility and solution properties [115]. Chitosan is the *N*-deacetylated derivative of chitin with a typical DA of less than 0.35, therefore being a copolymer composed of glucosamine and *N*-acetylglucosamine [121].

CH, CHs and its derivatives are widely applied in different economical sectors, such as agriculture [122], water treatment [123], food and cosmetic industry [124], pharmaceutical and medicine [125]. Properties that make natural CH and CHs attractive polymers for various applications, mainly in pharmaceutics and medicine, are their antimicrobial activity, film-forming ability, high adsorption, biodegrability, biocompatibility and non-toxicity [126]. Among biomedical applications reported for CH and CHs are tissue engineering, wound healing, drug delivery and cancer diagnosis [127].

The use of CHs in the food industry is related to its functional properties, principally water- and fat-binding capacity [126] as well as emulsifying properties [128]. Also the antimicrobial activity of CHs has been exploited for the preparation of films as a packaging material for a preservation of a variety of foods [129]. Besides, CHs microparticles are being evaluated as carriers for essential oils in cosmetic formulations [130].

## 6. Traditional CH and CHs Production Processes

The most common sources of CH are crab and shrimp shell wastes. In the skeletal tissue of these species, CH is bound to proteins forming a chitin-protein matrix associated to mineral salts [11], principally calcium carbonate. In this regard, the main components of crustacean shells are on a dry weight basis and depending on the species and season, 30%–40% protein, 30%–50% mineral salts and 13%–42% CH [28]. Furthermore, small amounts of lipids from muscle or viscera residues [117] and carotenoids, mainly astaxanthin and is esters [131], associated with proteins of the exoskeleton can be found in crustacean shell waste.

**Figure 3:**
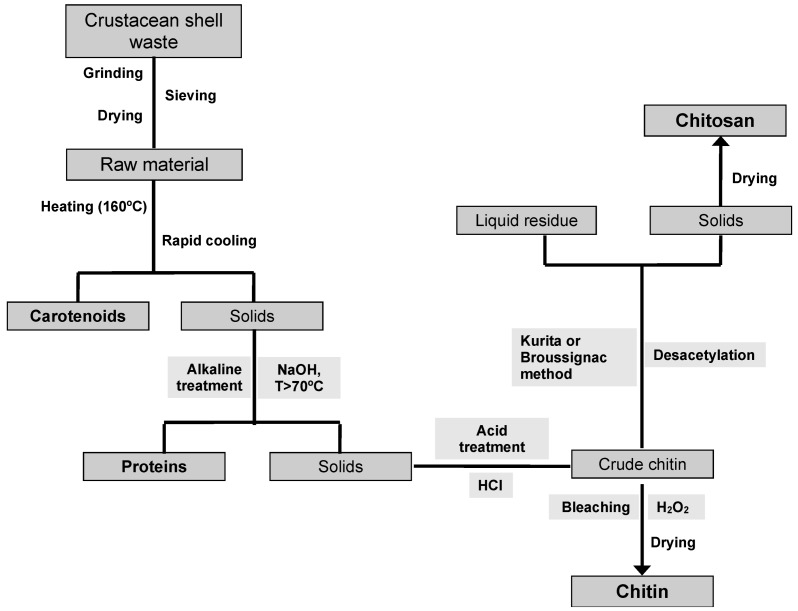
Scheme of CH and CHs preparation from crustacean shell waste using chemical methods.

The traditional method for the industrial recovery of CH from different crustacean shells consists of two steps (Figure 3), including a deproteinization with alkali treatment at high temperatures and a demineralization using diluted hydrochloric acid as the preferred reagent. Although it is considered that the order of these two phases is interchangeable depending on the source and proposed use of chitin [117], other authors suggest that demineralization should be performed first in order to decrease the residual mineral content [132].

After demineralization and deproteinization, CH isolated from crustacean sources has a lightly pink color and so a bleaching process using potassium permanganate, oxalic acid [133] or hydrogen peroxide [132] is usually carried out to yield a colorless product. On the contrary, CH isolated from squid pens is completely white and therefore, this final stage is unnecessary.

It is generally accepted that the processing conditions significantly affect the molecular weight and acetylation degree of CH. In this sense, the stronger the acidic conditions utilized for demineralization (pH, time and temperature), the lower molecular weight products are obtained [11]. Percot *et al.* studied the kinetics of demineralization of shrimp shells by following the pH variations in the reaction medium [11]. According to their results, they were able to define the optimal conditions necessary to perform a complete reaction, minimizing the hydrolysis of the glycosidic bonds. For this purpose, an excess of 0.25 M HCl, a solid-to-liquid ratio above 10 mL/g and 15 min of reaction at ambient temperature provided a final product with a DA above 95%.

In contrast, deproteinization by alkaline treatment has shown to be less damaging to the chitin structure compared to the acidic treatment involved in the demineralization [119]. In fact, Percot *et al.* reported that deproteinization using 1 M NaOH with a temperature and a reaction time below 70 °C and 24 h had no influence on both the molecular weight and DA, respectively [11]. Nevertheless, a large variation exists for the reported conditions of deproteinization for CH preparation. Chang and Tsai [134] analyzed protein removal from shrimp shell waste using NaOH by response surface methodology, reporting optimal conditions with 2.5 N NaOH, 75 °C and a minimal solution to solid ratio of 5 mL/g. According to their results, these authors also reported that kinetics of demineralization and deproteinization were pseudo-first order and two-stage first-order reactions, respectively. Tolaimate *et al.*, using a tailored isolation process according to the source of CH (shrimp, crab, lobster or squid), were able to obtain highly acetylated products (near 100%) preserving the crystalline structure of both α and β chitin [132]. These authors using low concentrated acid (0.55 M HCl) and base (0.3 M NaOH) solutions in a multi-stage process, highlighted the need to adapt the process conditions to the origin and specific characteristics of the CH source utilized.

On the other hand, the most commonly used methods for CHs production are the Broussignac [135] and Kurita [106] processes. The first procedure consists of a deacetylation of chitin in a nearly anhydrous reaction medium using a mixture of potassium hydroxide, ethanol and monoethylene glycol. On the other hand, the Kurita method proceeds in a stirred aqueous solution of sodium hydroxide, under a nitrogen stream at high temperatures (>80 °C).

Tolaimate *et al.* extensively compared both deacetylation methods and these studies indicated that the adjustment of different parameters related to the deacetylation process, the nature of the source, physical structure of the original CH and its isolation process allow to prepare CHs with controlled physico-chemical (molecular weight and DA) characteristics either from α or β-chitins [132,136]. Comparing the two processes for the production of CHs from a completely *N*-acetylated β-chitin prepared from squid pen (*Loligo vulgaris*), these authors concluded that the Kurita process enabled to obtain CHs with high molecular weights and a wide range of deacetylation degrees [136]. On the contrary, the Broussignac process could be carried out to obtain CHs with low degrees of acetylation and molecular weights, but in a faster way. Nevertheless, due to the high amounts of alkali and acid wastewaters generated in these production processes, there is a need to find alternatives to overcome the problem of wastewater neutralization. A possible way that has not been sufficiently explored to date is the reutilization of these effluents in the alkaline proteolysis step of CS isolation from cartilage (Figure 1). This strategy would allow the recycling of highly polluting wastewaters and goes towards the overall utilization of marine by-products.

## 7. Alternative CH and CHs Production Processes

**Figure 4:**
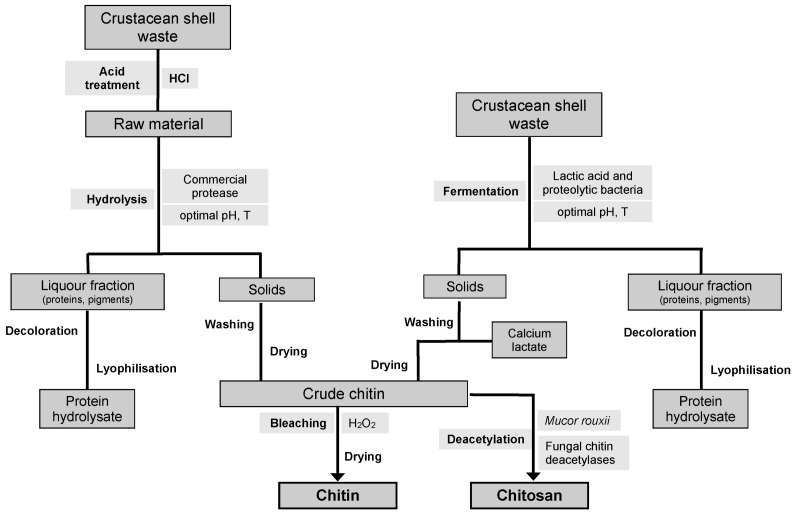
Scheme of chitin and chitosan preparation from crustacean shell waste using eco-friendly methods.

Chemical CH purification is an energy consuming process and results in environmental problems with high waste processing costs, due to the need of neutralization of processing wastewaters [111]. Besides and as stated above, prolonged alkaline and acid treatments cause depolymerization and deacetylation of the polysaccharide. Furthermore, the low biological value of alkali-recovered proteins may limit its application in the animal feed industry, thus affecting the production costs of CH and CHs from crustacean by-products [117]. In recent years, several methods have been reported in the literature to solve chemical extraction problems (Figure 4). One of the biological alternatives proposed is the use of proteases for deproteinization of crustacean shells, avoiding alkaline treatments. Various commercial proteases have been assayed for protein removal from crustacean shells [137], being alcalase the most employed and effective enzyme [137,138,139]. In addition, the utilization of crude proteolytic extracts obtained from different microorganisms [140,141] or even from fish viscera [142] have been studied, leading to varying deproteinization yields depending on the conditions assayed. Although deproteinization levels achieved in such cases are generally lower than those obtained using alkaline treatments, this alternative has the advantage to produce nutritionally valuable protein hydrolysates in addition to chitin [138].

When using enzymatic deproteinization, previous demineralization is more convenient since it increases the permeability of the tissues and reduces the presence of potential enzyme inhibitors, favoring the subsequent action of the enzyme [138].

Another biotechnological approach for the production of CH from seafood wastes consists on their fermentation using lactic acid bacteria (LAB). The production of bio-silages from fish by-products consists on the ability of LAB strains to ferment the waste materials and to produce *in situ* organic acids, mainly lactic and acetic acids, in order to preserve and produce ingredients for animal feed production [18,102]. This methodology has also been applied for the recovery of other value-added by-products from ensilaged shrimp waste, such as carotenoids [143].

In the fermentation of crustacean by-products, two fractions are obtained: a solid phase containing crude chitin and a liquor fraction rich in proteins, minerals and pigments. This occurs because lactic acid produced during fermentation operates at two levels. On the one hand, it reacts with the calcium carbonate to produce calcium lactate, which precipitates and can be easily removed by washing [144]. In addition, lactic acid decreases pH values, leading to the activation of proteases. Deproteinization of the biowaste and simultaneous liquefaction of the proteins occurs mainly by proteolytic enzymes produced by the added LAB, by gut bacteria of the intestinal system of crustaceans, or by proteases present in the source byproduct [145].

Several LAB have been assayed in a wide range of raw materials of marine origin. Shrimp waste has been mainly fermented using *Lactobacillus plantarum* [113,146], but also with other lactic acid bacteria such as *Lactobacillus paracasei* [147], *Pediococcus acidolactici* [148] and *Lactobacillus helveticus* [149]. Non-LAB, including *Pseudomonas aeruginosa* K-187 [150] and *Bacillus subtilis* [151] have been assayed as inoculum source for the recovery of CH. Commercial bacterial inoculums containing a mixture of LAB have been utilized for the production of CH from waste shell of prawn (*Nephrops norvegicus*). Stabisil containing *Streptococcus faecium* M74, *L. plantarum*, and *P. acidilactici* [152] and a powdered grass silage inoculant consisting of a mixture of selected proteolytic enzyme producing bacteria [107] proved to be effective alternatives for the demineralization and deproteinization of prawn biowastes.

Duan *et al.* reported the production of CH from shrimp waste by fermentation with the epiphytic strain *Lactobacillus acidophilus* SW01 isolated from shrimp by-products [133]. Due to its high protease activity, the solid residue from fermented shrimp waste contained less than 1% minerals and proteins. Therefore, after 168 h of cultivation at 37 °C, pure CH could be easily recovered only following a bleaching treatment.

Co-fermentation using a LAB and a bacterium with proteolytic activity has also been investigated as an alternative for CH purification from marine by-products. The LAB *Lactococcus lactis* and *Teredinobacter turnirae*, a protease producer marine bacterium, were jointly utilized for the for biological CH extraction from prawn waste [153]. Both bacteria were cultivated individually and co-fermented in a culture medium prepared with 10% (w/v) shell solids in the presence of increasing concentrations of glucose (0%–15% w/v). Although the extraction of CH following this procedure was incomplete compared to the chemical method, the highest process yield (95.5%) was obtained when *T. turnirae* was first inoculated in co-fermentation. Similar results were obtained by Jung *et al.*, who co-cultivated the lactic acid bacterium *L. paracasei* subsp. tolerans KCTC-3074 and the protease producing bacterium *Serratia marcescens* FS-3 in crab shells [154]. These authors founded that the co-fermentation process was efficient, although highlighted the need to improve deproteinization.

In a later paper, Jung *et al.* reported for the first time successive two-step fermentation from red crab shell wastes using the same species than in the previous work [111,154]. This research concluded that the sequential order of inoculation is an important issue, since the best results in co-removal of CaCO_3_ and proteins, 94.3% and 68.9%, respectively, from crab shells were obtained when successive fermentation was carried out in a first step with *S. marcescens* followed by a second cultivation with *L. paracasei*, and not *vice versa*.

Several process variables have been reported to influence the fermentation of marine wastes and therefore the efficiency of CH recovery from these sources, such as inoculum ratio [147], temperature [155] and initial pH [146]. Also carbon source and level, and the carbon on nitrogen ratio [147,156] were found to be important parameters for CH recovery from crustacean shells. Although the majority of the reports use the one-factor-at-a-time approach to study the effect of these variables on fermentation performances, other studies have attempted to optimize fermentation conditions for chitin recovery using response surface methodology [148,149,155].

Nevertheless, from the stated above it follows that demineralization and deproteinization occur simultaneously but incompletely in these biological processes [111]. This lower performance of LAB fermentation in deproteinization and demineralization of shell waste has been attributed to the compact structure of the shells [113]. For this reason, the fermentation of crustacean shells has been reported as a complementary strategy to chemical treatments, leading to a decrease in the amount of corrosive chemicals in the CH extraction process [112]. In addition to the reduction in the use of reagents, a major advantage of the fermentation process is obtaining a high-value by-product in the form of liquor rich in protein, minerals and asthaxanthin [113].

The same manner as chemical CH purification, the production of CHs by deacetylation of crustacean chitin with strong alkali appears to have limited potential for industrial acceptance, because of the large amounts of concentrated alkaline solution waste causing environmental pollution. Moreover, the conversion of CH to CHs, using a strong base solution at high temperature, causes variability of the product properties, decreases the CHs quality and increases the processing costs [157]. An alternative source of CHs is the cell wall of fungi, mainly zygomycetes. Among them, the fungus *M. rouxii* has been reported to contain significant amounts of CHs, CH and acidic polysaccharides as cell wall components [158]. For this reason, bioproduction of CHs from *M. rouxii* has been widely studied during recent years [117,159,160,161,162]. According to Chatterjee *et al.* culture media and fermentation conditions can be varied to provide CHs of more consistent physico-chemical properties compared to that obtained by chemical modification of chitin [159]. Among three fungal culture media, molasses salt medium (MSM), potato dextrose broth (PDB) and yeast extract peptone glucose (YPG), chitosan from MSM was less polydispersed and more crystalline compared to those from YPG and PDB, thus indicating a higher quality of the polymer.

Since CHs is a constituent of *M. rouxii* cell walls, its production is coupled to fungal growth, and therefore maximal productions are obtained when mycelial growth is maximal. CHs molecular weight was found to be dependent on the growth phase of *M. rouxii*, showing an increase of molecular weight with time of culture [160]. These authors also found a great influence of the pH on fungal growth and therefore on CHs production. Trutnau *et al.* found a higher CHs content with increasing time of cultivation in semi-continuous cultures, suggesting an adaption of the fungi to shear stress [162]. According to these authors, their results and model predictions of hyphal growth, suggest that repeated batch cultures might be optimal for CHs production.

Naturally occurring CHs is produced *in situ* by enzymatic deacetylation of chitin [163]. CH deacetylases were characterized in various fungi, such as *M. rouxii* [164], *Rhizopus nigricans* [165] and *Aspergillus nidulans* [166]. These enzymes have been also explored as an alternative to alkali treatment on chitin production from crustacean shells. Nevertheless, fungal CH deacetylases studied so far are only able to perform enzymatic deacetylation on their solid substrate to a 5%–10% of the total *N*-acetylglucosamine residues [167], preferring *N*-acetylglucosamine homopolymers as substrates [164]. Therefore pretreatment of crystalline CH would be necessary prior to enzyme hydrolysis, in order to improve the accessibility of acetyl groups to the enzyme. Several physical and chemical methods such as heating, sonicating, grinding, derivatization and interaction with saccharides have been assayed in order to improve the accessibility to the acetyl groups for the deacetylation [168]. Win and Stevens were successful at deacetylating CH to CHs (10% DA), using a chitin deacetylase from the fungus *Absidia coerulea* [167]*.* In this work a pretreatment of superfine CH, a decrystallized form with a very small particle size, with 18% formic acid resulted in the nearly complete enzymatic deacetylation.

Finally it is important to note that besides allowing the reduction in the use of chemicals, fungal CHs possesses two advantages that are interesting for medical applications: a lower molecular weight and lower contents of heavy metals [162]. In Table 2, different microbial processes studied for CH and CHs from marine sources are reported.

**Table 2:** Summary of procedures and conditions for CH and CHs productionfrom marine sources.

Final Product	Source	Procedure	Process conditions	Yield (*Y*)/Efficiency (*DM*, *DP*, *DD*)	Ref.
CH	prawn shell	anaerobic fermentation	Sil-Al 4 × 4 TM inoculant, glucose, 30 °C, 7 days	*DP* = 91%/*Y* = 20%	[107]
CH	red crab shell	successive two-step fermentation	*S. marcescens*, *L. paracasei*, glucose, 30 °C, 7 days	*DM* = 94.3%/*DP* = 68.9%/*Y* = 38.7%	[111]
CH	shrimp waste	anaerobic fermentation	*L. acidophilus* SW01, glucose, 37 °C, 168 h	*DM* = 99.3%/*DP* = 96.5%	[133]
CH	demineralised prawn shell	solid-state fermentation	Stabisil inoculant, lactose, 25 °C	*DP* = 40%	[152]
CH	prawn shell	co-fermentation	*L. lactis*, *T. turnirae*, glucose, 7 days	*DM* = 70%/*DP* = 70%/*Y* = 95.5%	[153]
CH	red crab shell	co-fermentation	*L. paracasei*, *S. marcescens*, glucose, 30 °C, 7 days	*DM* = 97.2%/*DP* = 52.6%	[154]
CHs	*M. rouxii*	semi-continuous fermentation	nutrient broth, 28 °C, 24 h	*DD =* 86%–88%/*Y =* 4.4%	[69]
CHs	*M. rouxii*	fermentation	*MSM*, *PDB*, *YPG*	*DD*(*MSM*) = 87.2%/*DD*(*PDB*) = 89.8%/*DD*(*YPG*) = 82.8%/*Y*(*MSM*) = 7.7%/*Y*(*PDB*) = 6%/*Y*(*YPG*) = 6.3%	[159]

*DM*, demineralization; *DP*, deproteinization; *DD*, deacetylation degree; *MSM*, molasses salt medium; *PDB*, potato dextrose broth; *YPG*, yeast extract peptone glucose.

## 8. Conclusions

CS, HA and CH/CHs have attracted increasing attention because of their beneficial effects on several ambits of the human health, in the formulation of cosmeceuticals and anti-aging products, nutraceuticals and food ingredients as well as their application in bio and nanotechnological processes. From long time ago, extensive studies have been conducted on the clarification of the general aspects of the chemical structures, features, novel applications and more sustainable processes for their production. In this review, we have discussed a set of recent progresses in the definition of eco-friendly processes to extract and purify those biomacromolecules from marine by-products.

## References

[1] Food and Agriculture Organization (2004). Estadísticas de Pesca: Captura y Desembarques.

[2] Gildberg A. (1992). Recovery of proteinases and protein hydrolysates from fish viscera. Bioresour. Technol..

[3] Blanco M., Sotelo C.G., Chapela M.J., Pérez-Martín R.I. (2007). Towards sustainable and efficient use of fishery resources: Present and future trends. Trends Food Sci. Technol..

[4] European Community On a European Community plan of action for the conservation and management of sharks. http://ec.europa.eu/fisheries/marine_species/wild_species/sharks/index_en.htm.

[5] Arvanitoyannis I.S., Kassaveti A. (2008). Fish industry waste: Treatments, environmental impacts, current and potential uses. Int. J. Food Sci. Technol..

[6] Senevirathne M., Kim S.-K. (2012). Utilization of seafood processing by-products. Medicinal applications. Adv. Food Nutr. Res..

[7] Jayathilakan K., Sultana K., Radhakrishna K., Bawa A.S. (2012). Utilization of byproducts and waste materials from meat, poultry and fish processing industries: A review. J. Food Sci. Technol..

[8] Kristinsson H.G., Rasco B. (2000). Fish protein hydrolysates: Production, biochemical and functional properties. Crit. Rev. Food Sci. Nutr..

[9] Gómez-Guillén M.C., Turnay J., Fernández-Díaz M.D., Ulmo N., Lizarbe M.A., Montero P. (2002). Structural and physical properties of gelatine extracted from different marine species: A comparative study. Food Hydrocoll..

[10] Gildberg A. (2004). Enzymes and bioactive peptides from fish waste related to fish silage, fish feed and fish sauce preparation. J. Aquat. Food Prod. Technol..

[11] Percot A., Viton C., Domard A. (2003). Optimization of chitin extraction from shrimp shells. Biomacromolecules.

[12] Vázquez J.A., González M.P., Murado M.A. (2004). A new marine medium. Use of different fish peatones and comparative study of selected species of marine bacteria. Enzym. Microb. Technol..

[13] Aspmo S.I., Horn S.J., Eijsink V.G.H. (2005). Hydrolysates from Atlantic cod (*Gadus morhua* L.) viscera as components of microbial growth media. Process Biochem..

[14] Vázquez J.A., Docasal S.F., Mirón J., González M.P., Murado M.A. (2006). Proteases production by two *Vibrio* species on residuals marine media. J. Ind. Microbiol. Biotechnol..

[15] Vázquez J.A., Docasal S.F., Prieto M.A., González M.P., Murado M.A. (2008). Growth and metabolic features of lactic acid bacteria in media with hydrolysed fish viscera. An approach to bio-silage of fishing by-products. Bioresour. Technol..

[16] Murado M.A., González M.P., Vázquez J.A. (2009). Recovery of proteolytic and collagenolytic activities from viscera by-products of rayfish (*Raja clavata*). Mar. Drugs.

[17] Giménez B., Alemám A., Montero P., Gómez-Guillén M.C. (2009). Antioxidant and functional properties of gelatine hydrolysates obtained from skin of sole and squid. Food Chem..

[18] Vázquez J.A., Nogueira M., Durán A., Prieto M.A., Rodríguez-Amado I., Rial D., González M.P., Murado M.A. (2011). Preparation of marine silage of swordfish, ray and shark visceral waste by lactic acid bacteria. J. Food Eng..

[19] Herpandi N.H., Adzitey F. (2011). Fish bone and scale as a potential source of halal gelatine. J. Fish. Aquat. Sci..

[20] Khaled H.B., Bougatef A., Balti R., Triki-Ellouz Y., Souissi N., Nasri M. (2008). Isolation and characterisation of trypsin from sardinelle (*Sardinella aurita*) viscera. J. Sci. Food Agric..

[21] Kim S.-B., Ji C.-I., Woo J.-W., Do J.-R., Cho S.-M., Lee Y.-B., Kang S.-N., Park J.-H. (2012). Simplified purification of chondroitin sulphate from scapular cartilage of shortfin mako shark (*Isurus oxyrinchus*). Int. J. Food Sci. Technol..

[22] Jayasinghe P., Hawboldt K. (2012). A review of bio-oils from waste biomass: Focus on fish processing waste. Renew. Sustain. Ener. Rev..

[23] Silva T.H., Alves A., Ferreira B.M., Oliveira J.M., Reys L.L., Ferreira R.J.F., Sousa R.A., Silva S.S., Mano J.F., Reis R.L. (2012). Materials of marine origin: A review on polymers and ceramics of biomedical interest. Int. Mat. Rev..

[24] Ronca F., Palmieri L., Panicucci P., Ronca G. (1998). Anti-inflammatory activity of chondroitin sulfate. Osteoarthr. Cartil..

[25] Kogan G., Soltés L., Stern R., Gemeiner P. (2007). Hyaluronic acid: A natural biopolymer with a broad range of biomedical and industrial applications. Biotechnol. Lett..

[26] Zou X.H., Jiang Y.Z., Zhang G.R., Jin H.M., Nguyen T.M., Ouyang H.W. (2009). Specific interactions between human fibroblasts and particular chondroitin sulphate molecules for wound healing. Acta Biomater..

[27] Kumar M.N.V.R. (2000). A review of chitin and chitosan applications. React. Funct. Polym..

[28] Kurita K. (2006). Chitin and chitosan: Functional biopolymers from marine crustaceans. Mar. Biotechnol..

[29] Mourya V.K., Inamdar N.N. (2008). Chitosan-modifications and applications: Opportunities galore. React. Funct. Polym..

[30] Seno N., Meyer K. (1963). Comparative biochemistry of skin the mucopolysaccharides of shark skin. Biochim. Biophys. Acta.

[31] Vieira R.P., Mourao P.A. (1988). Occurrence of a unique fucosebranched chondroitin sulfate in the body wall of a sea cucumber. J. Biol. Chem..

[32] Kinoshita-Toyoda A., Yamada S., Haslam S.M., Khoo K.-H., Sugiura M., Morris H.R., Dell A., Sugahara K. (2004). Structural determination of five novel tetrasaccharides containing 3-*O*-sulfated d-glucuronic acid and two rare oligosaccharides containing a β-d-glucose branch isolated from squid cartilage chondroitin sulfate E. Biochemistry.

[33] Hardingham T., Garg H.G., Hales C.A. (2004). Solution Properties of Hyaluronan. Chemistry and Biology of Hyaluronan.

[34] Malavaki C., Mizumoto S., Karamanos N., Sugahara K. (2008). Recent advance in the structural study of functional chondroitin sulfate and dermatan sulphate in health and disease. Connect. Tissue Res..

[35] Garnjanagoonchorn W., Wongekalak L., Engkagul A. (2007). Determination of chondroitin sulfate from different sources of cartilage. Chem. Eng. Proc..

[36] Lauder R.M. (2009). Chondroitin sulphate: A complex molecule with potential impacts on a wide range of biological systems. Complement. Ther. Med..

[37] Schiraldi C., Cimini D., de Rosa M. (2010). Production of chondroitin sulphate and chondroitin. Appl. Microbiol. Biotechnol..

[38] Pipitone V.R. (1991). Chondroprotection with chondroitin sulphate. Drugs Exp. Clin. Res..

[39] Chang C.H., Liu H.C., Lin C.C., Chou C.H., Lin F.H. (2003). Gelatin–chondroitin–hyaluronan tri-copolymer scaffold for cartilage tissue engineering. Biomaterials.

[40] Cai S., Liu Y., Shu X.Z., Prestwich G.D. (2005). Injectable glycosaminoglycan hydrogels for controlled release of human basic fibroblast growth factor. Biomaterials.

[41] Keskin D.S., Tezcaner A., Korkusuz P., Korkusuz F., Hasirei V. (2005). Collagen-chondroitin sulfate-based PLLA-SAIB-coated rhBMP-2 delivery system for bone repair. Biomaterials.

[42] Yamada S., Sugahara K. (2008). Potential therapeutic application of chondroitin sulfate/dermatan sulphate. Curr. Drug Discov. Technol..

[43] Alkhalil A., Achur R.N., Valiyaveettil M., Ockenhouse C.F., Gowda D.C. (2000). Structural requirements for the adherence of *Plasmodium falciparum*-infected erythrocytes to chondroitin sulphate proteoglycans of human placenta. J. Biol. Chem..

[44] Smetsers T.F., van de Westerlo E.M., ten Dam G.B., Overes I.M., Schalkwijik J., van Muijen G.N., van Kuppvelt T.H. (2004). Human single-chain antibodies reactive with native chondroitin sulphate detect chondroitin sulphate alterations in melanoma and psoriasis. J. Invest. Dermatol..

[45] Pothacharoen P., Siriaunkgul S., Ong-Chai S., Supabandhu J., Kumja P., Wanaphirak C., Sugahara K., Hardingham T., Kongtawelert P. (2006). Raised serum chondroitin sulphate epitope level in ovarian epithelial cancer. J. Biochem..

[46] Borsig L., Wang L., Cavalcante M.C., Cardilo-Reis L., Ferreira P.L., Mourao P.A., Esko J.D., Pvao M.S. (2007). Selectin blocking activity of a fucosylated chondroitin sulphate glycosaminoglycan from sea cucumber. Effect on tumor metastasis and neutrophil recruitment. J. Biol. Chem..

[47] Hamano T., Mitsuhashi Y., Acki N., Yamamoto S., Tsuji S., Ito Y., Oji Y. (1989). High-performance liquid chromatography assay of chondroitin sulfate in food products. Analyst.

[48] Conte A., Volpi N., Palmieri L., Bahous I., Ronca G. (1995). Biochemical and pharmacokinetic aspects of oral treatment with chondroitin sulphate. Arzneim. Forsch..

[49] Volpi N. (2009). Quality of different chondroitin sulphate preparations in relation to their therapeutic activity. J. Pharm. Pharmacol..

[50] Michel B.A., Stucki G., Frey D., de Vathaire F., Vignon E., Bruehlmann P., Uebelhart D. (2005). Chondroitins 4 and 6 sulfate in osteoarthritis of the knee: A randomized, controlled trial. Arthritis Rheum..

[51] Field I.C., Meekan M.G., Buckworth R.C., Bradshaw C.J.A. (2010). Susceptibility of sharks, rays and chimaeras to global extinction. Adv. Mar. Biol..

[52] García V.B., Lucifora L.O., Myers R.A. (2008). The importance of habitat and life history to extinction risk in sharks, skates, rays and chimaeras. Proc. R. Soc. B.

[53] Shiedlin A., Bigelow R., Christopher W., Arbabi S., Yang L., Maier R.V., Wainwright N., Childs A., Miller R.J. (2004). Evaluation of hyaluronan from different sources *Streptococcus zooepidemicus*, rooster comb, bovine vitreous, and human umbilical cord. Biomacromolecules.

[54] Beasley K.L., Weiss M.A., Weiss M.D. (2009). Hyaluronic acid fillers: A comprehensive review. Facial Plast. Surg..

[55] Chong B.F., Blank L.M., Mclaughlin R., Nielsen L.K. (2005). Microbial hyaluronic acid production. Appl. Microbiol. Biotechnol..

[56] Murado M.A., Montemayor M.I., Cabo M.L., Vázquez J.A., González M.P. (2012). Optimization of extraction and purification process of hyaluronic acid from fish eyeball. Food Bioprod. Proc..

[57] Kim S.J., Park S.Y., Kin C.W. (2006). A novel approach to the production of hyaluronic acid by *Streptococcus zooepidemicus*. J. Microbiol. Biotechnol..

[58] Rangaswamy V., Jain D. (2008). An efficient process for production and purification of hyaluronic acid from *Streptococcus equi* subsp.* zooepidemicus*. Biotechnol. Lett..

[59] Rodén L., Baker J.R., Cifonelli J.A., Mathews M.B. (1972). Isolation and characterization of connective tissue polysaccharides. Methods Enzymol..

[60] Chascall V., Calabro A., Midura R.J., Yanagishita M., Lennarz W.J., Hart G.W. (1994). Isolation and Characterization of Proteoglycans. Methods in Enzymology.

[61] Sumi T., Ohba H., Ikegami T., Shibata M., Sakaki T., Salay I., Park S.S. (2002). Method for the Preparation of Chondroitin Sulfate Compounds. U.S. Patent.

[62] Patel B., Ehrlich J., Stivala S.S., Singh N.K. (1980). Comparative studies of mucopolysaccharides from marine animals. I. *Raja eglanteria* Bosc. J. Exp. Mar. Biol. Ecol..

[63] Lignot B., Lahogue V., Bourseau P. (2003). Enzymatic extraction of chondroitin sulfate from skate cartilage and concentration-desalting by ultrafiltration. J. Biotechnol..

[64] Mollard L., Montillet A., Horriere C., Legrand J., Nguyen T.H. (2005). Method for Obtaining Avian Biological Products. U.S. Patent.

[65] Tadashi E. (2006). Sodium Chondroitin Sulfate,Chondroitin-Sulfate-Containing Material and Processes for Producing the Same. U.S. Patent Appl..

[66] Murado M.A., Fraguas J., Montemayor M.I., Vázquez J.A., González M.P. (2010). Preparation of highly purified chondroitin sulphate from skate (*Raja clavata*) cartilage by-products. Process optimization including a new procedure of alkaline hydroalcoholic hydrolysis. Biochem. Eng. J..

[67] Michelacci Y.M., Horton D.S.P.Q. (1989). Proteoglycans from the cartilage of young hammerhead shark *Sphyrna lewini*. Comp. Biochem. Physiol..

[68] Souza A.R.C., Kozlowski E.O., Cerqueira V.R., Castelo-Branco M.T.L., Costa M.L., Pavão M.S.G. (2007). Chondroitin sulfate and keratan sulfate are the major glycosaminoglycans present in the adult zebrafish *Danio rerio* (Chordata-Cyprinidae). Glycoconj. J..

[69] Gargiulo V., Lanzetta R., Parrilli M., de Castro C. (2009). Structural analysis of chondroitin sulfate from *Scyliorhinus canicula*: A useful source of this polysaccharide. Glycobiology.

[70] Takai M., Kono H. (2003). Salmon-Origin Chondroitin Sulphate. U.S. Patent Appl..

[71] Nishigori T., Takeda T., Ohori T. (2000). Method for Isolation and Purification of Chondroitin Sulfate. Jpn Patent.

[72] Jang H., Yoon Y.K., Kim J.A., Kim H.S., An S.J., Seo J.H., Cui C., Carbis R. (2008). Optimization of Vi capsular polysaccharide production during growth of *Salmonella enterica* serotype Typhi Ty2 in a bioreactor. J. Biotechnol..

[73] Cimini D., de Rosa M., Schiraldi C. (2012). Production of glucuronic acid-based polysaccharides by microbial fermentation for biomedical applications. Biotechnol. J..

[74] Rimler R.B. (1994). Presumptive identification of *Pasteurella multocida* serogroups A, D and F by capsule depolymerisation with mucopolysaccharides. Vet. Rec..

[75] Rimler R.B., Register K.B., Magyar T., Ackermann M.R. (1995). Influence of chondroitinase on indirect hemagglutination titers and phagocytosis of *Pasteurella multocida* serogroups A, D and F. Vet. Microbiol..

[76] Cimini D., Restaino O.F., Catapano A., de Rosa M., Schiraldi C. (2010). Production of capsular polysaccharide from *Escherichia coli* K4 for biotechnological applications. Appl. Microbiol. Biotechnol..

[77] Restaino O.F., Cimini D., de Rosa M., Catapano A., de Rosa M., Schiraldi C. (2011). High cell density cultivation of *Escherichia coli* K4 in a microfiltration bioreactor: A step towards improvement of chondroitin precursor production. Microb. Cell Fact..

[78] Schiraldi C., Alfano A., Cimini D., de Rosa M., Panariello A., Restaino O.F., de Rosa M. (2012). Application of a 22L scale membrane bioreactor and cross-flow ultrafiltration to obtain purified chondroitin. Biotechnol. Prog..

[79] Schiraldi C., Carcarino L.I., Alfano A., Restaino O.F., Panariello A., de Rosa M. (2011). Purification of chondroitin precursor from *Escherichia coli* K4 fermentation broth using membrane processing. Biotechnol. J..

[80] Bedini E., de Castro C., de Rosa M., di Nola A., Iadonisi A., Restaino O.F., Schiraldi C., Parrilli M. (2011). A microbiological-chemical strategy to produce chondroitin sulphate A,C. Angew. Chem. Int. Ed..

[81] Nakano T., Nakano K., Sim J.S. (1994). A simple rapid method to estimate hyaluronic acid concentrations in rooster comb and wattle using cellulose acetate electrophoresis. J. Agric. Food Chem..

[82] Cullis-Hill D. (1989). Preparation of Hyaluronic Acid from Synovial Fluid. U.S. Patent.

[83] Marcellin E., Chen W., Nielsen L.K., Rehm B.H.A. (2009). Microbial Hyaluronic Acid Biosynthesis. Microbial Production of Biopolymers and Polymer Precursors: Applications and Perspectives.

[84] Imberty A., Lortat-Jacobb H., Pérez S. (2007). Structural view of glycosaminoglycan–protein interactions. Carbohydr. Res..

[85] Swann D.A. (1968). Studies on hyaluronic acid: I. The preparation and properties of rooster comb hyaluronic acid. Biochim. Biophys. Acta.

[86] Balazs E.A. (1979). Ultrapure Hyaluronic Acid and the Use Thereof. U.S. Patent.

[87] O’Regan M., Martini I., Crescenzi F., de Luca C., Lansing M. (1994). Molecular mechanisms and genetics of hyaluronan biosynthesis. Int. J. Biol. Macromol..

[88] Radulescu G., Lupescu I., Petrea D.-M., Scurei H. (1997). Hyaluronic acid extraction from vitreous fluid. Biotechnol. Lett..

[89] Gao Y.-Q., Liu J.-H., Huo X., Shan Y.-L., Xu Z.-X. (1996). The purification and identification of hyaluronic acid isolated from various tissues. Chin. Boichem. J..

[90] Mizuno H., Iso N., Saito T., Ogawa H., Sawairi H., Saito M. (1991). Characterization of hyaluronic acid of yellowfin tuna eyeball. Nippon Suisan Gakkaishi.

[91] Ogrodowski C.S., Hokka C.O., Santana M.H.A. (2005). Production of hyaluronic acid by *Streptococcus*: The effects of the addition of lysozyme and aeration on the formation and the rheological properties of the product. Appl. Biochem. Biotechnol..

[92] Liu L., Du G., Chen J., Wang M., Sun J. (2008). Influence of hyaluronidase addition on the production of hyaluronic acid by batch culture of *Streptococcus zooepidemicus*. Food Chem..

[93] Johns M.R., Goh L.T., Oeggerli A. (1994). Effect of pH, agitation and aeration on hyaluronic acid production by *Streptococcus zooepidemicus*. Biotechnol. Lett..

[94] Huang W.C., Chen S.J., Chen T.L. (2006). The role of dissolved oxygen and function of agitation in hyaluronic acid fermentation. Biochem. Eng. J..

[95] Liu L., Du G., Chen J., Wang M., Sun J. (2009). Comparative study on the influence of dissolved oxygen control approaches on the microbial hyaluronic acid production of *Streptococcus zooepidemicus*. Bioprocess Biosyst. Eng..

[96] Hiruta O., Yamamura K., Takebe H., Futamura T., Ilnuma K., Tanaka H. (1997). Application of maxblend fermentor for microbial processes. J. Ferm. Bioeng..

[97] Liu L., Wang M., Du G., Chen J. (2008). Enhanced hyaluronic acid production of *Streptococcus zooepidemicus* by an intermittent alkaline-stress strategy. Lett. Appl. Microbiol..

[98] Blank L.M., McLaughlin R.L., Nielsen L.K. (2005). Stable production of hyaluronic acid in *Streptococcus zooepidemicus* chemostats operated at high dilution rate. Biotechnol. Bioeng..

[99] Zhang J., Ding X., Yang L., Kong Z. (2006). A serum-free medium for colony growth and hyaluronic acid production by *Streptococcus zooepidemicus* NJUST01. Appl. Microbiol. Biotechnol..

[100] Liu L., Du G., Chen J., Wang M., Sun J. (2008). Enhanced hyaluronic acid production by a two-stage culture strategy based on the modeling of batch and fed-batch cultivation of *Streptococcus zooepidemicus*. Bioresour. Technol..

[101] Vázquez J.A., Montemayor M.I., Fraguas J., Murado M.A. (2009). High production of hyaluronic and lactic acids by *Streptococcus zooepidemicus* in fed-batch culture using commercial and marine peptones from fishing by-products. Biochem. Eng. J..

[102] Vázquez J.A., Montemayor M.I., Fraguas J., Murado M.A. (2010). Hyaluronic acid production by *Streptococcus zooepidemicus* in marine by-products media from mussel processing wastewaters and tuna peptone viscera. Microb. Cell Fact..

[103] Yamada T., Kawasaki T. (2005). Microbial synthesis of hyaluronan and chitin: New approaches. J. Biosci. Bioeng..

[104] Teng W.L., Khor E., Tan T.K., Lim L.Y., Tan S.C. (2001). Concurrent production of chitin from shrimp shells and fungi. Carbohydr. Res..

[105] Roca C., Chagas B., Farinha I., Freitas F., Mafra L., Aguiar F., Oliveira R., Reis M.A.M. (2012). Production of yeast chitin-glucan complex from biodiesel industry byproduct. Process Biochem..

[106] Kurita K., Tomita K., Tada T., Ishii S., Nishimura S.-I., Shimoda K. (1993). Squid chitin as a potential alternative chitin source: Deacetylation behavior and characteristic properties. J. Polym. Sci. A.

[107] Healy M., Green A., Healy A. (2003). Bioprocessing of marine crustacean shell waste. Acta Biotechnol..

[108] Jo G.H., Jung W.J., Kuk J.H., Oh K.T., Kim Y.J., Park R.D. (2008). Screening of protease-producing *Serratia*
*marcescens* FS-3 and its application to deproteinization of crab shell waste for chitin extraction. Carbohydr. Polym..

[109] Manni L., Ghorbel-Bellaaj O., Jellouli K., Younes I., Nasri M. (2010). Extraction and characterization of chitin, chitosan, and protein hydrolysates prepared from shrimp waste by treatment with crude protease from *Bacillus cereus* SV1. Appl. Biochem. Biotechnol..

[110] Bautista J., Jover M., Gutiérrez J.F., Corpas R., Cremades O., Fontiveros E., Iglesias F., Vega J. (2001). Preparation of crayfish chitin by *in situ* lactic acid production. Process Biochem..

[111] Jung W.J., Jo G.H., Kuk J.H., Kim Y.J., Oh K.T., Park R.D. (2007). Production of chitin from red crab shell waste by successive fermentation with *Lactobacillus*
*paracasei* KCTC-3074 and *Serratia*
*marcescens* FS-3. Carbohydr. Polym..

[112] Cira L.A., Huerta S., Hall G.M., Shirai K. (2002). Pilot scale lactic acid fermentation of shrimp wastes for chitin recovery. Process Biochem..

[113] Rao M.S., Stevens W.F. (2005). Chitin production by *Lactobacillus* fermentation of shrimp biowaste in a drum reactor and its chemical conversion to chitosan. J. Chem. Technol. Biotechnol..

[114] Muzzarelli R.A.A. (1977). Chitin.

[115] Rinaudo M. (2006). Chitin and chitosan: Properties and applications. Prog. Polym. Sci..

[116] Minke R., Blackwell J. (1978). The structure of β-chitin. J. Mol. Biol..

[117] Synowiecki J., Al-Khateeb N.A. (2003). Production, properties, and some new applications of chitin and its derivatives. Crit. Rev. Food Sci. Nutr..

[118] Tharanathan R.N., Kittur F.S. (2003). Chitin—The undisputed biomolecule of great potential. Crit. Rev. Food Sci. Nutr..

[119] Roberts G.A.F. (1992). Chitin Chemistry.

[120] Rutherford F.A., Austin P.R., Muzzarelli R.A.A., Pariser E.R. (1978). Marine Chitin Properties and Solvents. Proceedings of the First International Conference on Chitin/Chitosan.

[121] Pillai C.K.S., Paul W., Sharma C.P. (2009). Chitin and chitosan polymers: Chemistry, solubility and fiber formation. Prog. Polym. Sci..

[122] Campaniello D., Bevilacqua A., Sinigaglia M., Corbo M.R. (2008). Chitosan: Antimicrobial activity and potential applications for preserving minimally processed strawberries. Food Microbiol..

[123] Huang D., Wang W., Kang Y., Wang A. (2012). Chitin and chitosan as multipurpose natural polymers for groundwater arsenic removal and As_2_O_3_ delivery in tumor therapy. J. Macromol. Sci. Pure Appl. Chem..

[124] Sánchez R., Stringari G.B., Franco J.M., Valencia C., Gallegos C. (2011). Use of chitin, chitosan and acylated derivatives as thickener agents of vegetable oils for bio-lubricant applications. Carbohydr. Polym..

[125] Aam B.B., Heggset E.B., Norberg A.L., Sørlie M., Vârum K.M., Eijsink V.G.H. (2010). Production of chitooligosaccharides and their potential applications in medicine. Mar. Drugs.

[126] Zhang J., Xia W., Liu P., Cheng Q., Tahirou T., Gu W., Li B. (2010). Chitosan modification and pharmaceutical/biomedical applications. Mar. Drugs.

[127] Jayakumar R., Menon D., Manzoor K., Nair S.V., Tamura H. (2010). Biomedical applications of chitin and chitosan based nanomaterials—A short review. Carbohydr. Polym..

[128] Rodríguez M.S., Albertengo L.A., Agulló E. (2002). Emulsification capacity of chitosan. Carbohydr. Polym..

[129] Fajardo P., Martins J.T., Fuciños C., Pastrana L., Teixeira J.A., Vicente A.A. (2010). Evaluation of a chitosan-based edible film as carrier of natamycin to improve the storability of Saloio cheese. J. Food Eng..

[130] Anchisi C., Meloni M.C., Maccioni A.M. (2006). Chitosan beads loaded with essential oils in cosmetic formulations. J. Cosmet. Sci..

[131] Shahidi F., Synowiecki J. (1991). Isolation and characterization of nutrients and value-added products from snow crab (*Chionoecetes opilio*) and shrimp (*Pandalus borealis*) processing discards. J. Agric. Food Chem..

[132] Tolaimate A., Desbrières J., Rhazi M., Alagui A. (2003). Contribution to the preparation of chitins and chitosans with controlled physico-chemical properties. Polymers.

[133] Duan S., Li L., Zhuang Z., Wu W., Hong S., Zhou J. (2012). Improved production of chitin from shrimp waste by fermentation with epiphytic lactic acid bacteria. Carbohydr. Polym..

[134] Chang K.L.B., Tsai G. (1997). Response surface optimization and kinetics of isolating chitin from pink shrimp (*Solenocera melantho*) shell waste. J. Agric. Food Chem..

[135] Broussignac P. (1968). Unhaut polymere naturel peu connu dans l’industrie, Le chitosane. Chim. Ind. Genie Chim..

[136] Tolaimate A., Desbrières J., Rhazi M., Alagui A., Vincendon M., Vottero P. (2000). On the influence of deacetylation process on the physicochemical characteristics of chitosan from squid chitin. Polymer.

[137] Valdez-Peña A.U., Espinoza-Pérez J.D., Sandoval-Fabian G.C., Balagurusamy N., Hernández-Rivera A., De-la-Garza-Rodríguez I.M., Contreras-Esquivel J.C. (2010). Screening of industrial enzymes for deproteinization of shrimp head for chitin recovery. Food Sci. Biotechnol..

[138] Synowiecki J., Al-Khateeb N.A.A.Q. (2000). The recovery of protein hydrolysate during enzymatic isolation of chitin from shrimp *Crangon crangon* processing discards. Food Chem..

[139] De Holanda H.D., Netto F.M. (2006). Recovery of components from shrimp (*Xiphopenaeus kroyeri*) processing waste by enzymatic hydrolysis. J. Food Sci..

[140] Giyose N.Y., Mazomba N.T., Mabinya L.V. (2010). Evaluation of proteases produced by *Erwinia chrysanthemi* for the deproteinization of crustacean waste in a chitin production process. Afr. J. Biotechnol..

[141] Haddar A., Hmidet N., Ghorbel-Bellaaj O., Fakhfakh-Zouari N., Sellami-Kamoun A., Nasri M. (2011). Alkaline proteases produced by *Bacillus licheniformis* RP1 grown on shrimp wastes: Application in chitin extraction, chicken feather-degradation and as a dehairing agent. Biotechnol. Bioprocess Eng..

[142] Sila A., Nasri R., Bougatef A., Nasri M. (2012). Digestive alkaline proteases from the goby (*Zosterisessor ophiocephalus*): Characterization and potential application as detergent additive and in the deproteinization of shrimp wastes. J. Aquat. Food Prod. Technol..

[143] Sachindra N.M., Bhaskar N., Siddegowda G.S., Sathisha A.D., Suresh P.V. (2007). Recovery of carotenoids from ensilaged shrimp waste. Bioresour. Technol..

[144] Hoffmann K., Daum G., Köster M., Kulicke W.-M., Meyer-Rammes H., Bisping B., Meinhardt F. (2010). Genetic improvement of *Bacillus licheniformis* strains for efficient deproteinization of shrimp shells and production of high-molecular-mass chitin and chitosan. Appl. Environ. Microbiol..

[145] Woods B. (1998). Microbiology of Fermented Foods.

[146] Rao M.S., Muñoz J., Stevens W.F. (2000). Critical factors in chitin production by fermentation of shrimp biowaste. Appl. Microbiol. Biotechnol..

[147] Shirai K., Guerrero I., Huerta S., Saucedo G., Castillo A., Gonzalez R.O., Hall G.M. (2001). Effect of initial glucose concentration and inoculation level of lactic acid bacteria in shrimp waste ensilation. Enzym. Microb. Technol..

[148] Bhaskar N., Suresh P.V., Sakhare P.Z., Sachindra N.M. (2007). Shrimp biowaste fermentation with *Pediococcus acidolactici* CFR2182: Optimization of fermentation conditions by response surface methodology and effect of optimized conditions on deproteination/demineralization and carotenoid recovery. Enzym. Microb. Technol..

[149] Arbia W., Adour L., Amrane A., Lounici H. (2013). Optimization of medium composition for enhanced chitin extraction from *Parapenaeus longirostris* by *Lactobacillus helveticus* using response surface methodology. Food Hydrocoll..

[150] Ghorbel-Bellaaj O., Jellouli K., Younes I., Manni L., Ouled S.M., Nasri M. (2011). A solvent-stable metalloprotease produced by *Pseudomonas aeruginosa* A2 grown on shrimp shell waste and its application in chitin extraction. Appl. Biochem. Biotechnol..

[151] Sini T.K., Santhosh S., Mathew P.T. (2007). Study on the production of chitin and chitosan from shrimp shell by using *Bacillus subtilis* fermentation. Carbohydr. Res..

[152] Healy M.G., Romo C.R., Bustos R. (1994). Bioconversion of marine crustacean shell waste. Resour. Conserv. Recycl..

[153] Aytekin O., Elibol M. (2010). Cocultivation of *Lactococcus lactis* and *Teredinobacter turnirae* for biological chitin extraction from prawn waste. Bioprocess Biosyst. Eng..

[154] Jung W.J., Jo G.H., Kuk J.H., Kim K.Y., Park R.D. (2006). Extraction of chitin from red crab shell waste by cofermentation with *Lactobacillus paracasei* subsp. tolerans KCTC-3074 and *Serratia marcescens* FS-3. Appl. Microbiol. Biotechnol..

[155] Pacheco N., Garnica-González M., Ramírez-Hernández J.Y., Flores-Albino B., Gimeno M., Bárzana E., Shirai K. (2009). Effect of temperature on chitin and astaxanthin recoveries from shrimp waste using lactic acid bacteria. Bioresour. Technol..

[156] Choorit W., Patthanamanee W., Manurakchinakorn S. (2008). Use of response surface method for the determination of demineralization efficiency in fermented shrimp shells. Bioresour. Technol..

[157] Synowiecki J., Al-Khateeb N.A.A.Q. (1997). Mycelia of *Mucor rouxii* as a source of chitin and chitosan. Food Chem..

[158] Arcidiacono S., Kaplan D.L. (1992). Molecular weight distribution of chitosan isolated from *Mucor rouxii* under different culture and processing conditions. Biotechnol. Bioeng..

[159] Chatterjee S., Adhya M., Guha A.K., Chatterjee B.P. (2005). Chitosan from *Mucor rouxii*: Production and physico-chemical characterization. Process Biochem..

[160] Chatterjee S., Chatterjee B.P., Guha A.K. (2010). Kinetics of *Mucor rouxii* fermentation in relation to chitosan production. Res. J. Microbiol..

[161] Martinou A., Bouriotis V., Stokke B.T., Vårum K.M. (1998). Mode of action of chitin deacetylase from *Mucor rouxii* on partially *N*-acetylated chitosans. Carbohydr. Res..

[162] Trutnau M., Suckale N., Groeger G., Bley T., Ondruschka J. (2009). Enhanced chitosan production and modeling hyphal growth of *Mucor rouxii* interpreting the dependence of chitosan yields on processing and cultivation time. Eng. Life Sci..

[163] Kolodziejska I., Malesa-Ciecwierz M., Lerska A., Sikorski Z. (1999). Properties of chitin deacetylase from crude extracts of *Mucor rouxii* mycelium. J. Food Biochem..

[164] Araki Y., Ito E. (1974). A pathway of chitosan formation in *Mucor rouxii*: Enzymatic deacetylation of chitin. Biochem. Biophys. Res. Commun..

[165] Jeraj N., Kunic B., Lenasi H., Breskvar K. (2006). Purification and molecular characterization of chitin deacetylase from *Rhizopus nigricans*. Enzyme Microb. Technol..

[166] Alfonso C., Nuero O.M., Santamaria F., Reyes F. (1995). Purification of a heat-stable chitin deacetylase from *Aspergillus nidulans* and its role in cell wall degradation. Curr. Microbiol..

[167] Win N.N., Stevens W.F. (2001). Shrimp chitin as substrate for fungal chitin deacetylase. Appl. Microbiol. Biotechnol..

[168] Zhao Y., Park R.-D., Muzzarelli R.A.A. (2010). Chitin deacetylases: Properties and applications. Mar. Drugs.

